# Treatment of Symptomatic Vitreomacular Adhesion with Expansile Sulfur Hexafluoride (SF6) Gas

**DOI:** 10.2174/1874364101711010080

**Published:** 2017-04-28

**Authors:** Dominic M Buzzacco, Sugat S Patel

**Affiliations:** 1 Havener Eye Institute, The Ohio State University, 915 Olentangy River Road, Columbus, Ohio, 43212, USA; 2 Midwest Retina, 6655 Post Road, Columbus, Ohio, 43016, USA

**Keywords:** Intravitreal, Vitreomacular, Adhesion, Expansile, Diabetes, Sulphur hexaflouride

## Abstract

**Background:**

To evaluate intravitreal injection of expansile sulfur hexafluoride (SF6) as a low cost and effective treatment for symptomatic vitreomacular adhesion (sVMA).

**Methods:**

Retrospective analysis of all patients with sVMA treated with pneumatic vitreolysis using SF6 gas presenting to a clinical practice from January 2005 and June 2013.

**Results:**

Six cases were included in the study. Five patients (83%) experienced complete resolution of the vitreomacular adhesion. One patient had a partial release of the vitreomacular adhesion. Four patients (67%) had a one line improvement in best corrected visual acuity.

**Conclusion:**

Due to its low cost, wide availability, and apparent efficacy, intravitreal injection of expansile SF6 should be investigated further as a possible treatment modality for sVMA.

## INTRODUCTION

With age, the human vitreous undergoes liquefaction and separation from the retina [1]. Occasionally, vitreous attachment to the macula is abnormally strong causing traction, macular edema, or a macular hole. This condition has come to be known as symptomatic vitreomacular adhesion (sVMA) [2]. Herein is a retrospective interventional case series of 6 patients who underwent treatment of sVMA by pneumatic vitreolysis with intravitreal injection of expansile sulfur hexafluoride (SF6).

## REPORT OF CASES

**Fig. (1):**
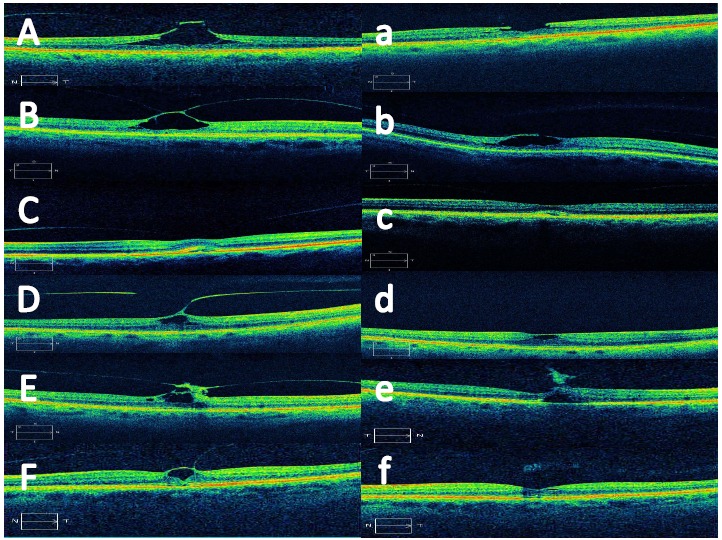
Pre-operative SD-OCT images of patients 1 through 6 (A-F) and SD-OCT images following pneumatic vitreolysis in patients 1 through 6 (a-f).

*Case 1.* A 78 year old phakic female with diabetes without retinopathy presented with blurred vision and distortion in the left eye. Best-corrected visual acuity (BCVA) at presentation was 20/60 in the left eye. Retinal examination showed mottling of the retinal pigmented epithelium (RPE) in the left eye. Spectral domain optical coherence tomography (SD-OCT) imaging of the macula confirmed vitreomacular adhesion (VMA) with a central foveal thickness of 391 um (Fig. **1A**) and no evidence of epiretinal membrane (ERM). The patient was treated with an intravitreal injection of 0.3 cc 100% sulfur hexafluoride (SF6) and concurrent anterior chamber paracentesis. At 1 week, the VMA had released and CFT on SD-OCT had improved to 253 um Fig. (**1a**). A lamellar macular defect was also noted on SD-OCT. Best corrected visual acuity remained stable at 20/60.

*Case 2.* An 84 year old pseudophakic female with diabetes mellitus without retinopathy and low risk age related macular degeneration (ARMD) presented with blurred vision in the right eye. BCVA was 20/200 in the right eye. Retinal examination showed the clinical appearance of a stage 1b hole. SD-OCT of the macula confirmed VMA with a stage 1b macular hole (Fig. **1B**) and no evidence of ERM. The patient was treated with an intravitreal injection of 0.3 cc 100% SF6 and concurrent anterior chamber paracentesis. At 1 month, SD-OCT revealed partial release of the VMA with development of a lamellar defect (Fig. **1b**). Central foveal thickness improved to 252 um and BCVA acuity improved to 20/100.

*Case 3.* A 76 year old phakic female with low risk ARMD presented with blurred vision in the left eye. BCVA was 20/50 in the left eye. Retinal examination showed mild thickening and RPE mottling in the macula. SD-OCT of the macula confirmed VMA with central foveal thickness of 277 um (Fig. **1C**) and no evidence of ERM. The patient was treated with an intravitreal injection of 0.3 cc 100% SF6 and concurrent anterior chamber paracentesis. At 1 month, SD-OCT revealed resolution of the VMA and central foveal thickness of 252 um (Fig. **1c**). BCVA improved to 20/40.

*Case 4.* A 76 year old phakic female with mild non-proliferative diabetic retinopathy presented with blurred vision in the right eye. BCVA was 20/40 in the right eye. Retinal examination revealed an ERM and RPE mottling. SD-OCT of the macula confirmed the ERM with VMA (Fig. **1D**). Central foveal thickness was 385 um. The patient was treated with an intravitreal injection of 0.3 cc 100% SF6 and concurrent anterior chamber paracentesis. At 2 weeks, SD-OCT showed release of the VMA with central foveal thickness of 326 um (Fig. **1d**). BCVA improved to 20/30 at 2 weeks and 20/25 at 9 months.

*Case 5.* A 58 year old phakic female presented with blurred vision in the right eye. She had a history of proliferative diabetic retinopathy and diabetic macular edema. BCVA was 20/40 in the right eye. Retinal examination revealed exudates and cystic changes with preretinal gliosis in the macula. SD-OCT confirmed VMA with central foveal thickness of 306 um (Fig. **1E**). The patient was treated with an intravitreal injection of 0.3 cc 100% SF6 and concurrent anterior chamber paracentesis. At 1 month, SD-OCT showed release of the VMA with central foveal thickness of 248 um (Fig. **1e**). BCVA improved to 20/40.

*Case 6.* A 62 year old phakic female presented with blurred vision in the left eye. She had low risk ARMD. The patient also had history of a previous macular hole in the right eye. BCVA was 20/50 in the right eye. Retinal examination showed the clinical appearance of a stage 1a macular hole. SD-OCT of the macula confirmed VMA with a stage 1a hole (Fig. **1F**) and no evidence of ERM. Central foveal thickness was 304 um. The patient was treated with an intravitreal injection of 0.3 cc 100% SF6 and concurrent anterior chamber paracentesis. At 1 month, SD-OCT revealed release of the VMA and central foveal thickness of 258 um (Fig. **1f**). BCVA improved to 20/40.

## COMMENT

The search for innovative ways to treat cases of sVMA has led to a number of options. Despite the success of vitrectomy surgery for sVMA, it does carry with it the inherent risk of any intraocular procedure including cataract progression, glaucoma, retinal detachment, and infection. More recently, ocriplasmin was met with enthusiasm as it provided an office based treatment option for symptomatic patients who wished to avoid vitrectomy. However, while more effective than placebo, patients in the MIVI-TRUST trial had only a 26% resolution of the adhesion when treated with ocriplasmin [3].

Early reports of injection of expansile gas in treating sVMA have been promising. Chan et al initially reported promising outcomes in treating macular holes with expansile gas induced vitreous separation [4]. Rodrigues et al. reported a 40% success rate at 1 month in treating sVMA with expansile perfluoropropane (C3F8) [5]. Certainly, the low cost and wide availability of these agents would make them a good treatment option should they prove efficacious.

In this series, five of the six (83%) patients had a complete resolution of the vitreomacular adhesion. Although one patient only had partial resolution of the VMA, there was a substantial decrease in central foveal thickness on OCT. Four patients (67%) had a 1 line improvement in visual acuity. Complications such as post procedure intraocular pressure increase, retinal tears, or cataract progression were not noted in this series.

## CONCLUSION

Overall, pneumatic vitreolysis with expansile gas seems to be a promising treatment option for patients with sVMA. To our knowledge, this is the first report of the use of SF6 gas in the treatment of sVMA. Further evaluation in randomized controlled trials seems warranted given the low cost, acceptable safety profile, and apparent efficacy.

## References

[1] Foos R.Y., Wheeler N.C. (1982). Vitreoretinal juncture. Synchysis senilis and posterior vitreous detachment.. Ophthalmology.

[2] Jackson T.L., Nicod E., Simpson A., Angelis A., Grimaccia F., Kanavos P. (2013). Symptomatic vitreomacular adhesion.. Retina.

[3] Stalmans P., Benz M.S., Gandorfer A., Kampik A., Girach A., Pakola S., Haller J.A., MIVI-TRUST Study Group (2012). Enzymatic vitreolysis with ocriplasmin for vitreomacular traction and macular holes.. N. Engl. J. Med..

[4] Chan C.K., Wessels I.F., Friedrichsen E.J. (1995). Treatment of idiopathic macular holes by induced posterior vitreous detachment.. Ophthalmology.

[5] Rodrigues I.A., Stangos A.N., McHugh D.A., Jackson T.L. (2013). Intravitreal injection of expansile perfluoropropane (c(3)f(8)) for the treatment of vitreomacular traction.. Am. J. Ophthalmol..

